# Association Between Cardiovascular Diseases and Lingual Varices: A Systematic Review and Meta-Analysis

**DOI:** 10.3390/jcm15166495

**Published:** 2026-08-21

**Authors:** Aya El Kanfoud-Ezzarraa, Sonia Egido-Moreno, Ani Mantinyan-Hakobyan, Mónica Blázquez Hinarejos, Beatriz González-Navarro, Anna Oliveras Serrano, Eva Otero-Rey, José López-López

**Affiliations:** 1 Department of Odontostomatology, Faculty of Medicine and Health Sciences, School of Dentistry, University of Barcelona, L’Hospitalet de Llobregat, 08907 Barcelona, Spain; aelkanez24@alumnes.ub.edu (A.E.K.-E.); amatinma8@alumnes.ub.edu (A.M.-H.); monicablazquez@ub.edu (M.B.H.); beatrizgonzalez@ub.edu (B.G.-N.); 2 Barcelona Dental Hospital [HOUB], 08970 Barcelona, Spain; 3 Department of Nephrology, Hospital del Mar, Pompeu Fabra University, 08003 Barcelona, Spain; aoliveras@hospitaldelmar.cat; 4 Oral Medicine, Oral Surgery and Implantology Unit, MedOralRes Group, University of Santiago de Compostela, 15782 Santiago de Compostela, Spain; evam.otero@usc.es

**Keywords:** lingual varices, cardiovascular diseases, oral vascular lesions, hypertension, cardiovascular risk, oral manifestations

## Abstract

**Background/Objectives**: Cardiovascular diseases (CVD) remain the leading cause of mortality worldwide. Early identification of individuals at risk is essential to reduce disease burden. Lingual varices (LV), a common oral vascular condition, have been suggested as a potential non-invasive clinical marker of systemic vascular alterations. This study aimed to evaluate the association between lingual varices and cardiovascular diseases through a systematic review and meta-analysis. **Methods**: A systematic review was conducted according to PRISMA guidelines and Cochrane recommendations and registered in PROSPERO (CRD420251172737). Electronic searches were performed in PubMed, Web of Science, and Scopus databases. Observational studies assessing the association between lingual varices and cardiovascular diseases in adults were included. Odds ratios (OR) with 95% confidence intervals (CI) were calculated, and meta-analysis was performed using fixed- or random-effects models depending on heterogeneity. **Results**: Thirteen studies involving 6322 participants were included. The overall prevalence of lingual varices was 34.8%. The meta-analysis demonstrated a significant association between lingual varices and cardiovascular disease (OR = 4.54; 95% CI: 3.35–6.17). Among the cardiovascular conditions evaluated, hypertension also showed a significant association with lingual varices (OR = 4.14; 95% CI: 1.66–10.29). Lingual varices were also significantly associated with smoking (OR = 3.06; 95% CI: 1.61–5.82) and lower extremity varicose veins (OR = 1.64; 95% CI: 1.04–2.58). No significant association was observed with sex (OR = 0.95; 95% CI: 0.67–1.34; *p* = 0.77). **Conclusions**: Lingual varices are significantly associated with cardiovascular disease. Although they may represent a readily identifiable oral finding associated with cardiovascular risk profile, the available evidence does not support their use as an independent screening or diagnostic marker. Further prospective studies are required to determine their predictive value and clinical utility.

## 1. Introduction

Cardiovascular diseases (CVDs) are the leading cause of mortality worldwide, accounting for approximately 20.5 million deaths each year. According to the World Health Organization’s International Classification of Diseases, 11th Revision (ICD-11), these include disorders of the heart and blood vessels such as hypertension, ischemic heart disease, heart failure, arrhythmias, and valvular diseases. It is worth noting that cerebrovascular diseases, such as stroke, are classified in ICD-11 under the chapter of nervous system diseases, although they share many risk factors with CVDs. Among these conditions, coronary heart disease and stroke account for more than 80% of deaths, and one third of these occur prematurely in individuals under 70 years of age [1,2,3].

CVDs remain the leading cause of death in Spain. In 2018, they accounted for 28.3% of all deaths, with a crude mortality rate of 258.6 deaths per 100,000 inhabitants, surpassing cancer (26.4%) and respiratory diseases (12.6%). CVDs were the leading cause of death among women (272.5 per 100,000 inhabitants) and the second leading cause among men (244.3 per 100,000 inhabitants) [4]. In Catalonia, a population-based study including 6,332,228 adults estimated a prevalence of 3.8% for ischemic heart disease, 3.8% for cerebrovascular disease, 3.3% for atrial fibrillation, and 2.5% for heart failure. In the same population, hypertension affected 24.6% of adults. Other cardiovascular risk factors included hyperlipidemia (20.6%), smoking (20.9%), obesity (18.5%), and diabetes mellitus (9.3%) [3,5].

Regarding the evolution of diabetes mellitus (DM) prevalence worldwide, a significant increase is observed in the most recent estimates. However, in the most recent update (10th edition, 2021), current estimates have risen to 537 million adults, with a projection of 783 million by 2045 [6,7]. Regarding the global burden of hypertension, a substantial increase has been observed over recent decades. According to the World Health Organization, approximately 1.28 billion adults aged 30–79 years were living with hypertension in 2019, and its burden is expected to continue increasing in the coming decades due to population aging and the persistence of associated cardiovascular risk factors [8].

In addition to their high mortality, CVDs represent a significant burden of morbidity and disability worldwide. Furthermore, the global cost associated with the treatment and management of CVDs is expected to continue rising due to population aging, urbanization, and the persistence of modifiable risk factors. In this context, early identification of individuals at higher cardiovascular risk is a public health priority [9,10]. In 2021, CVDs were estimated to cost the European Union €282 billion annually, accounting for 11% of total healthcare expenditure. In Spain, the total economic burden of cardiovascular diseases was estimated at approximately €24 billion in 2021, including healthcare, long-term care, productivity losses, and informal care costs [11,12].

Lingual varices (LV), also referred to as sublingual varicosities, are defined as abnormally dilated and tortuous veins. They are the most common type of acquired oral vascular lesion and are generally visible on the ventral surface and lateral borders of the tongue; less frequently, they may also be found on the lips and buccal mucosa. LV are the most common type of oral varicosity, present in approximately two-thirds of individuals over 60 years of age. Clinically, they appear as multiple raised, blue-purple, irregular or papular formations, distributed bilaterally from the posterior region of the ventral surface to the tip of the tongue. Most lesions are asymptomatic and are detected incidentally during routine clinical examinations [13].

Although LV are one of the most common acquired oral vascular lesions, there is no universally accepted standardized classification in the scientific literature for their diagnosis or clinical categorization. The only clinically used classification described in the literature for LV is that proposed by Hedström and Bergh (2010) [14], which consists of a simple dichotomous system based on clinical inspection of the ventral or lateral surface of the tongue. It distinguishes two grades: grade 0, when no varices are present or only a few discrete veins are observed, and grade 1, when varices are moderate or severe, with more numerous, dilated, and clearly visible veins. This system allows for rapid and standardized assessment, although it has the limitation of being insufficiently detailed, as it groups different levels of severity into a single category.

The development of LV is primarily associated with age, being rare in children and common in older adults, possibly linked to age-related degeneration and a decrease in the tone of the connective tissue surrounding the veins. Other predisposing factors include hypertension (HTN), DM, smoking, use of dental prostheses, and a history of varicose veins in the lower limbs. Histologically, venous dilation with a thin muscular wall and poor development of elastic tissue is observed; in cases of secondary thrombosis, the lumen may contain platelet and erythrocyte aggregates, and older thrombi may calcify, forming phleboliths [13].

Although LV have traditionally been considered a benign condition of limited clinical relevance, recent studies suggest that they may be associated with systemic diseases, particularly cardiovascular conditions. Various factors shared with CVDs may influence the development of LV. Alterations in blood vessels in one region of the body may reflect circulatory problems in other areas, indicating that both CVDs and LV could act as markers of generalized vascular dysfunction [15].

Aging impairs the mechanical and structural properties of the vascular wall, increasing susceptibility to circulatory problems, while chronic inflammation, both systemic and localized, has been associated with CVD and may contribute to the development of varices in different locations [14,16].

Collectively, these observations suggest that LV may represent a visible oral finding associated with cardiovascular disease. However, whether this association reflects an independent relationship or is largely explained by shared factors, such as ageing and other cardiovascular comorbidities, remains uncertain.

## 2. Materials and Methods

This systematic review was conducted in accordance with the Cochrane Handbook for Systematic Reviews and complies with the Preferred Reporting Items for Systematic Reviews and Meta-Analyses (PRISMA 2020 checklist in Supplementary Material File S2) statement [17]. The protocol was registered in PROSPERO (CRD420251172737). The clinical question addressed was whether an association exists between the presence of lingual varices (LV) and cardiovascular disease (CVD) in adults.

The PECO framework was defined as follows: adult individuals assessed for the presence or absence of lingual varices and cardiovascular outcomes (Population), presence of lingual varices (Exposure), absence of lingual varices (Comparison), and cardiovascular disease (CVD) (Outcome). For the purposes of this review, hypertension (HTN) was analyzed separately because it was consistently reported as an independent outcome in most of the included studies, whereas all other cardiovascular conditions were grouped under the overarching category of CVD whenever they were reported as a composite cardiovascular outcome by the original authors.

Eligible studies included cohort and cross-sectional studies that evaluated the association between LV and CVD and reported sufficient data to calculate odds ratios (ORs).

In vitro studies, animal studies, and studies that did not differentiate between patients with and without LV were excluded.

A comprehensive literature search was conducted in MEDLINE (via PubMed), Web of Science, and Scopus to identify relevant studies according to the predefined eligibility criteria. Study selection was performed in three stages: removal of duplicates using Mendeley Reference Manager (v2.144.0, Elsevier Ltd., Amsterdam, The Netherlands), screening of titles and abstracts based on the research question and eligibility criteria, and full-text assessment. The search strategy was based on key MeSH terms such as “varicose veins”, “lingual”, and “cardiovascular diseases”, combined using the Boolean operators AND and OR. The PubMed strategy included combinations of terms related to CVDs with terms related to lingual veins and varicosities; similar strategies were adapted for Web of Science and Scopus.

In addition, reference lists of the included studies were manually screened to identify any additional relevant articles not captured in the initial search. The methodological quality and risk of bias of the included studies were independently assessed by the reviewers.

The Newcastle–Ottawa Scale (NOS) was used for cohort studies [18,19], while the Joanna Briggs Institute (JBI) Critical Appraisal Tool was applied for cross-sectional studies [20,21].

Data extraction was performed using a structured table including author and reference, study design, country, total sample size, recruitment settings, sampling strategy, participation rate and age of participants (with distribution across comparison groups when available). Information on systemic association factors such as DM, hypercholesterolemia, metabolic syndrome, smoking, alcohol consumption, obesity, or body mass index was also collected, along with the use of relevant medications (e.g., antihypertensive or antidiabetic drugs). The presence of LV and a history of cardiovascular and cerebrovascular diseases were recorded, and, when available, systolic and diastolic blood pressure values were extracted. Continuous variables (e.g., age, body mass index, and blood pressure) were collected for descriptive purposes only and were not included in the quantitative synthesis because they were reported inconsistently across studies.

As all outcomes included in the quantitative synthesis were dichotomous, odds ratios (ORs) with corresponding 95% confidence intervals (95% CIs) were used as the summary measure of association.

When ORs were not directly reported, they were calculated from the raw frequencies or, when available, from percentages and corresponding sample sizes. Studies were excluded only when insufficient data were available to reconstruct a 2 × 2 contingency table.

Meta-analyses were performed using the Mantel–Haenszel method. Model selection considered both statistical heterogeneity and the expected clinical and methodological differences among the included studies, such as variations in study populations, diagnostic criteria, and outcome assessment. Fixed-effect models were initially applied when no substantial statistical heterogeneity was detected, whereas random-effects models were used when considerable heterogeneity was present. Statistical heterogeneity was assessed using Cochran’s Q test and the I^2^ statistic.

Statistical heterogeneity was assessed using Cochran’s Q test and the I^2^ statistic, with I^2^ values > 50% were considered indicative of substantial heterogeneity. All statistical analyses were conducted using Review Manager, version 5.4.1 (The Cochrane Collaboration, London, UK).

The certainty of evidence for the primary meta-analysed outcome (association between lingual varices and cardiovascular disease) was assessed using the Grading of Recommendations Assessment, Development and Evaluation (GRADE) framework [22]. Two reviewers independently assessed the certainty of evidence across the five GRADE domains: risk of bias, inconsistency, indirectness, imprecision, and publication bias. Disagreements were resolved by discussion and, when necessary, by consultation with a third reviewer.

As all included studies were observational, the certainty of evidence started at low according to the GRADE framework. The certainty of evidence could be downgraded by one or two levels for serious or very serious concerns regarding any of the five domains. Risk of bias was evaluated according to the methodological quality assessment performed using the Joanna Briggs Institute Critical Appraisal Checklist for Analytical Cross-Sectional Studies and the Newcastle–Ottawa Scale for cohort studies. Inconsistency was assessed considering the magnitude of statistical heterogeneity (I^2^ statistic) together with clinical and methodological heterogeneity across studies. Indirectness was evaluated by examining the applicability of the study populations, exposure, comparator, and outcomes to the review question. Imprecision was assessed according to the width of the confidence intervals, the number of included studies, and the overall robustness of the pooled estimates. Publication bias was considered when sufficient studies were available to permit formal assessment and was otherwise judged qualitatively.

The certainty of evidence could also be upgraded when appropriate according to GRADE criteria, including a large magnitude of effect, evidence of a dose–response gradient, or when all plausible residual confounding would reduce rather than explain the observed association.

## 3. Results

### 3.1. Search Results

**Figure 1:**
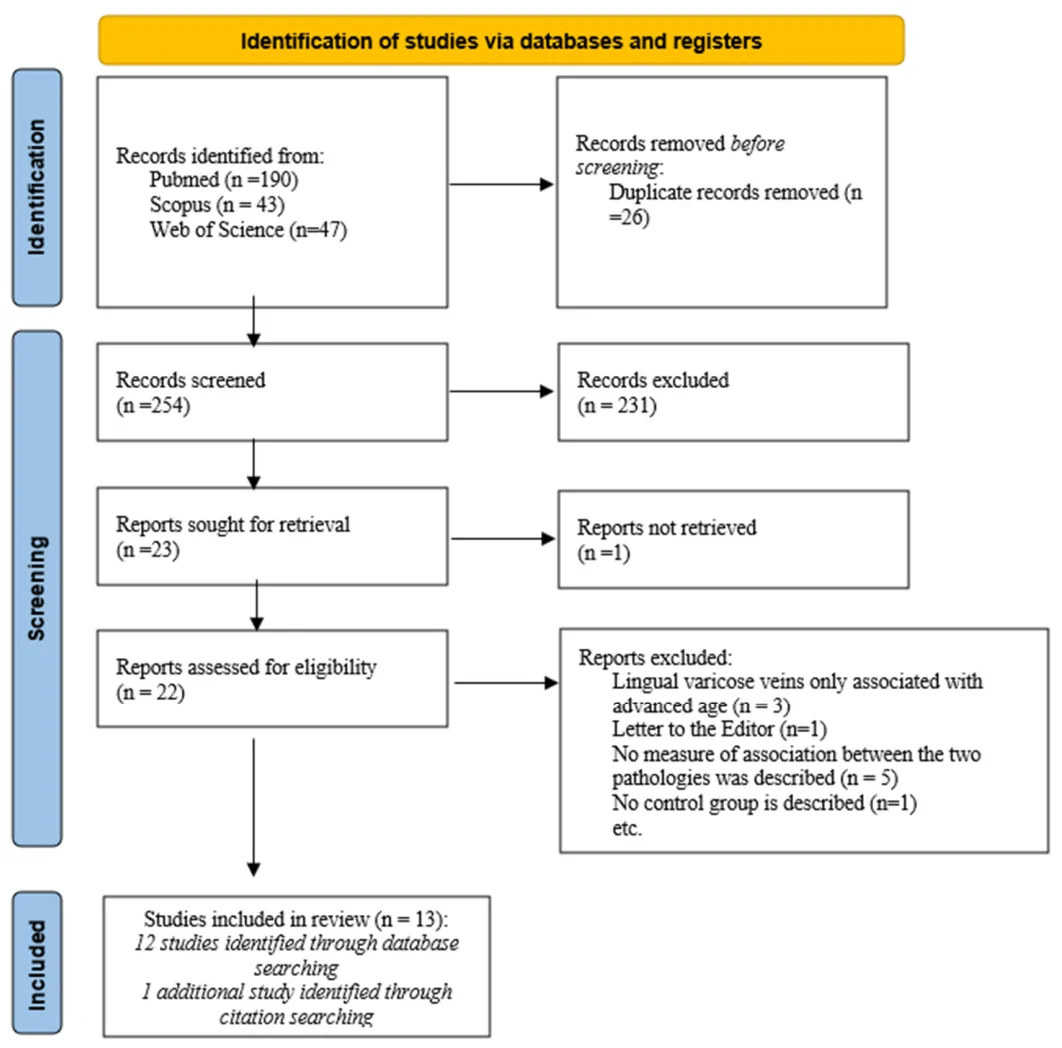
PRISMA 2020 flow diagram for systematic reviews and meta-analyses.

The initial literature search identified a total of 280 records across the selected databases: PubMed (*n* = 190), Scopus (*n* = 43), and Web of Science (*n* = 47). After removing duplicates (*n* = 26), 254 records remained for screening (Figure 1).

The titles and abstracts of these records were examined, and 231 were excluded for not addressing the research question. Subsequently, 23 articles were selected for full-text review, of which 1 could not be retrieved.

Finally, 22 full-text studies were assessed, and those that did not meet the eligibility criteria were excluded: three studies for focusing solely on lingual varices (LV) associated with advanced age [23,24,25], one for being a letter to the editor [26], five for not reporting prevalence or measures of association (odds ratio; OR) between the variables studied [27,28,29,30,31], and one for lacking a control group [32].

Twelve studies were identified through the electronic database search. One additional eligible study was identified through citation searching, resulting in a total of 13 studies included in the systematic review (Figure 1). Studies were identified that evaluated the association between LV and cardiovascular-associated factors. The study designs mainly included 11 cross-sectional studies (CS) and 2 cohort studies (CH).

### 3.2. Demographic Data

Based on the analyzed studies, the total accumulated sample comprised 6322 patients from diverse geographic settings. Regarding sex distribution, there was a slight female predominance, with 3555 women (W; 56%) compared to 2767 men (M; 44%) (Table 1a).

In terms of age, the study population mainly consisted of middle-aged and older adults. A weighted mean age of 58.6 years with a standard deviation of 14.6 was calculated, considering only those studies that provided complete data on mean and standard deviation (SD). In this regard, the study by Jafari et al. [33] was excluded due to the absence of SD data, in order to maintain methodological consistency in the analysis. Some studies included broader age ranges (from adolescents to elderly patients).

Regarding geographical origin, the studies were mainly conducted in Europe (especially in Sweden [14,34,35,36,37] and Italy [38]) and the Middle East (Iran [33,39,40] and Jordan [41]), as well as including populations from Turkey [42], India [43], and Brazil [44], reflecting a heterogeneous and multicultural sample. However, a greater representation of the Swedish population stands out, due to the higher number of studies conducted in this country.

Participants were predominantly recruited from dental care settings, including public and private dental clinics, university dental hospitals, oral medicine departments, and oral pathology services. One study was conducted in a geriatric outpatient clinic, another in nursing homes, and one retrospective study identified eligible cases through medical records and pathology archives. Consecutive sampling was the most frequently used recruitment strategy (*n* = 5), followed by convenience sampling (*n* = 5), whereas two studies employed census sampling and one retrospective study used archived clinical records. Participation rates were inconsistently reported across the included studies. When available, they ranged from 65.2% to 96.8%. The lowest participation rate was observed in one longitudinal study in which recruitment was prematurely interrupted by the COVID-19 pandemic, while several studies did not report participation rates or reasons for non-participation. These differences in recruitment setting and sampling strategy may have contributed to the heterogeneity observed across the included studies and should be considered when interpreting the pooled estimates (Table 1a).

**Table 1:** (**a**) General characteristics of the included studies, including study type, country, sample size, recruitment setting, sampling strategy and participation rate, sex distribution, and participants’ age. (**b**) Methodological characteristics of the included studies, including method of LV assessment, examiner reliability, and cardiovascular outcome assessment. Prepared by the authors based on the included studies.

(**a**)								
**Author**	**Study**	**Country**	**Population** **(Patients)**	**Recruitment Setting**	**Sampling Strategy**	**Participation Rate**	**Sex**	**Age (Years)**
Hedström and Bergh (2010) [14]	CS	Sweden	281	Public Dental Services	Consecutive sample of adult patients attending annual dental follow-up visits	NR	140 W 141 M	56 ± 12 (40–92)
Hedström et al. (2015) [34]	CS	Sweden	431	Public Dental Services	Consecutive sample of eligible dental patients attending routine recall visits	431/454 (94.9%) completed the study; total dropout 5.1% (23/454)	243 W 188 M	55.3 ± 10.9
Al-Shayyab and Baqain (2015) [41]	CS	Jordan	391	Department of Dentistry	Convenience sample of patients attending the dental department	NR	188 W 203 M	43.2 ± 14.6 (13–74)
Akkaya et al. (2019) [42]	CS	Türkiye	691	Department of Dentomaxillofacial Radiology	Convenience sample of patients attending for comprehensive clinical examination	691/714 (96.8%) participated; 23/714 (3.2%) excluded	470 W 221 M	38.88 ± 16 (18–88)
Shivakumar et al. (2020) [43]	CS	India	201	Krishna Hospital, Karad	Convenience sample of dental patients aged >40 years attending the hospital	201/215 (93.5%); 14 participants dropped out	80 W 121 M	52.3 ± 11.5
Accardo et al. (2021) [38]	CS	Italy	1008	Ambulatory of Pathophysiology for Elderly	Consecutive sample	NR	592 W 416 M	67.3 ± 2.7 (45–85)
Bergh et al. (2022a) [35]	CS	Sweden	989	Two dental clinics (one public and one private)	Consecutive sample	External participation rate not reported; internal dropout 11.7%	539 W 450 M	66.5 ± 7.4 (55–84)
Baharvand et al. (2022) [39]	CS	Iran	151	School of Dentistry	Convenience sample (volunteer dental patients)	NR	74 W 77 M	47.58 ± 12.19 (30–75)
Bergh et al. (2022b) [36]	CH	Sweden	281	Public Dental Services	Consecutive sample of eligible patients attending annual dental examinations	281/431 (65.2%); recruitment ended prematurely due to the COVID-19 pandemic	153 W 128 M	63.1 ± 9.9
Jafari et al. (2022) [33]	CS	Iran	478	Two nursing homes	Census sampling	478/509 (93.9%); 31 residents excluded due to conditions such as dementia or paralysis	276 W 202 M	74.5 (≥60)
Ahadian et al. (2023) [40]	CS	Iran	120	Department of Oral Medicine	Census sampling of eligible patients attending routine dental visits	NR	65 W 55 M	45.2 ± 15.1 (18–79)
Bergh et al. (2024) [37]	CH	Sweden	1139	Two dental clinics (one public and one private)	Consecutive sample of patients attending annual dental check-ups	1139/1420 (80.2%); 281 participants excluded or lost to follow-up	619 W 520 M	66.4 ± 7.3 (40–84)
Dos Santos et al. (2024) [44]	CS	Brazil	161	Oral Pathology Laboratory and Discipline of Oral Propaedeutics	Retrospective convenience sample based on medical records and pathology archives	NR	116 W 45 M	57.4 ± 16.4
(**b**)								
**Author**		**Method of LV Assessment**		**Examiner Reliability**	**Cardiovascular Outcome Assessment**			
Hedström and Bergh (2010) [14]		Direct clinical examination		NR	Self-reported medical history according to ICD-10 and medication history			
Hedström et al. (2015) [34]		Direct clinical examination		NR	Physician-confirmed diagnosis, participant self-report, antihypertensive medication history, and standardized blood pressure measurements performed during the study			
Al-Shayyab and Baqain (2015) [41]		Direct clinical examination		NR	Participant self-reported medical history classified according to ICD-10			
Akkaya et al. (2019) [42]		Direct clinical examination		NR	Participant self-reported medical history, including hypertension, other cardiovascular diseases, diabetes mellitus, smoking status, and lower extremity varicose veins.			
Shivakumar et al. (2020) [43]		Direct clinical examination		NR	Participant self-reported medical history and standardized blood pressure measurements performed during the study; hypertension was confirmed by repeated measurements in hospital.			
Accardo et al. (2021) [38]		Direct clinical examination		NR	Standardized office blood pressure measurements confirmed by 24 h ambulatory blood pressure monitoring (ABPM), together with clinical evaluation, medical history, and antihypertensive medication review.			
Bergh et al. (2022a) [35]		Standardized digital photographs		Two calibrated examiners	Physician-diagnosed cardiovascular diseases and risk factors obtained by questionnaire, together with standardized blood pressure measurements, anthropometric assessment, fasting blood tests, and medication history.			
Baharvand et al. (2022) [39]		Direct clinical examination		NR	Standardized blood pressure measurements performed during the study according to National Heart, Lung, and Blood Institute guidelines; participant health questionnaire.			
Bergh et al. (2022b) [36]		Standardized digital photographs		Two calibrated examiners	Physician-diagnosed cardiovascular conditions and risk factors obtained by participant questionnaire.			
Jafari et al. (2022) [33]		Direct clinical examination		NR	Standardized blood pressure measurements based on participants’ weekly medical records from routine nursing home assessments.			
Ahadian et al. (2023) [40]		Direct clinical examination		Oral medicine specialist	Standardized blood pressure measurements performed by an oral medicine specialist using two measurements according to the JNC7 classification.			
Bergh et al. (2024) [37]		Standardized digital photographs		Two blinded examiners; Cohen’s κ = 0.59	Physician-confirmed diagnoses obtained from participant questionnaires and validated using medical records; cardiovascular risk factors were assessed through questionnaires, medical records, anthropometric measurements, and laboratory tests.			
Dos Santos et al. (2024) [44]		Direct clinical examination		NR	Medical records, participant medical history, and standardized blood pressure measurements performed during the study.			

CS: Cross sectional; CH: Cohort; W: Women; M: Men. NR: Not reported.

### 3.3. Presence of LV and CVD

Across the analyzed studies, a total of 2200 cases of LV were identified (including all moderate/severe categories or total cases when not specified) out of 6322 patients (34.80%), highlighting the high frequency of this condition in the studied samples. In general, LV showed a variable distribution depending on the population, although in most studies mild or absent cases predominated over moderate/severe ones (Table 2a).

According to the included studies, 2565 participants had cardiovascular diseases (CVD) including hypertension (HTN), angina pectoris (AP), ischaemic heart disease (IHD), myocardial infarction (MI), and stroke, according to the definitions used in the original studies. Of these, 1210 also presented LV, corresponding to 47.2% of individuals with CVD. However, these data should be interpreted with caution due to the heterogeneity of the included studies (Table 2a).

HTN emerged as one of the most relevant CVD, with significant associations reported in multiple studies (OR between 1.61 and 2.30; *p* < 0.01) [39,43]. The presence of LV was also significantly associated with different forms of HTN, including controlled and resistant HTN (*p* < 0.005) [34,35,42]. However, some studies did not find statistical significance, reporting *p*-values > 0.05 (e.g., *p* = 0.891 [36] or *p* = 0.184 [44]), indicating no association in those specific samples.

Because the included studies used different clinical classifications for LV, all grading systems were harmonized into a dichotomous variable for quantitative synthesis. LV classified as grade 0 (G0), none, or mild were considered the reference category, whereas lesions classified as moderate, severe, grade 1 (G1) according to the Hedström and Bergh [14] classification, or equivalent categories indicating clinically evident LV were considered the exposed group. When studies already reported dichotomous classifications, the original definitions were retained.

The assessment of LV was predominantly performed by direct clinical examination, which was used in 10 of the 13 included studies. Three studies have assessed LV using standardized digital photographs of the ventral surface of the tongue [35,36,37]. Information regarding examiner reliability was limited and inconsistently reported. Most studies did not provide details on examiner training, calibration, or interobserver and intraobserver agreement. Two studies reported that the assessment was performed by two calibrated examiners [35,36], while one study additionally specified that the examiners were blinded and reported moderate interobserver agreement (Cohen’s κ = 0.59) [37]. One study indicated that the oral examination was performed by an oral medicine specialist [40], whereas the remaining studies did not report information on examiner reliability (Table 1b).

### 3.4. Factors Associated with LV

The presence of LV was highly associated with aging, showing a significant association in most studies (*p* < 0.001) [35,42], with odds ratios (OR) generally greater than 1 (ranging from 1.03 to 3.21) [36,38].

The presence of LV was also associated with smoking in several studies, with OR values ranging approximately from 2.01 to 3.13 [37,38] and statistically significant results (*p* < 0.001) [35,37,41]. However, some studies did not find a significant association or even reported trends without a clear relationship [33,36], highlighting the heterogeneity in the findings.

Regarding sex, the presence of LV was contradictory across studies. Some studies found no significant association (*p* ≥ 0.05) [35] or reported OR values close to 1 (OR = 0.79; *p* = 0.44) [14], suggesting no relationship. In contrast, other studies identified a higher odds among women (OR up to 2.74–2.89; *p* = 0.001) [38,42], while some reported a stronger association in men (OR = 2.07; *p* < 0.001) [36,41]. Overall, these results reflect considerable heterogeneity.

The presence of LV was significantly associated with other cardiovascular and metabolic factors (OR = 4.01; *p* < 0.001) [41]. In contrast, DM showed inconsistent evidence, with divergent results across studies (OR = 1.80; *p* < 0.05) [35,44] and (*p* = 0.517) [36].

An association between the presence of LV and dyslipidemia (DL) was reported (OR = 0.78; *p* = 0.168) [35], suggesting a possible lack of relationship, although not statistically significant. Conversely, the study by Accardo et al. [38] reported a statistically significant association between DL and LV (*p* < 0.05), although without providing a specific OR.

The presence of LV was also significantly associated with the use of removable denture (URD) in several studies (OR between 2.03 and 2.17; *p* < 0.05) [33,41,42]. Similarly, lower extremity varicose veins (LEV) were significantly associated with the presence of LV (*p* < 0.0001) [33].

Other factors, such as body mass index (BMI), did not show a significant association (*p* = 0.374) [36], suggesting no relationship with the condition in the analyzed population. However, an elevated waist-to-hip ratio showed a significant association (OR = 2.06; *p* = 0.004) [35], as did antihypertensive therapy (*p* < 0.05) [38]. These variables were less frequently assessed and showed occasional but non-uniform associations across studies.

In the prospective cohort studies, participants with LV showed a higher incidence of subsequent IHD and cerebrovascular events during follow-up [37]. In these contexts, age and smoking remained significantly associated factors (*p* < 0.0001) [37].

Overall, age was the factor most consistently associated with the presence of LV, followed by smoking and, to a lesser extent, other systemic and local variables.

### 3.5. Medication

Regarding medication, most of the studies analyzed did not provide specific information on patients’ pharmacological treatments. The study by Dos Santos et al. [44] reported patients’ medication use; however, it did not assess its influence in the multivariate analysis (Table 2).

Only the study by Accardo et al. evaluated antihypertensive therapy. No statistically significant association between antihypertensive treatment and lingual varices was observed [38].

### 3.6. Prevalence of LV and CVDs by Age Group

**Figure 2:**
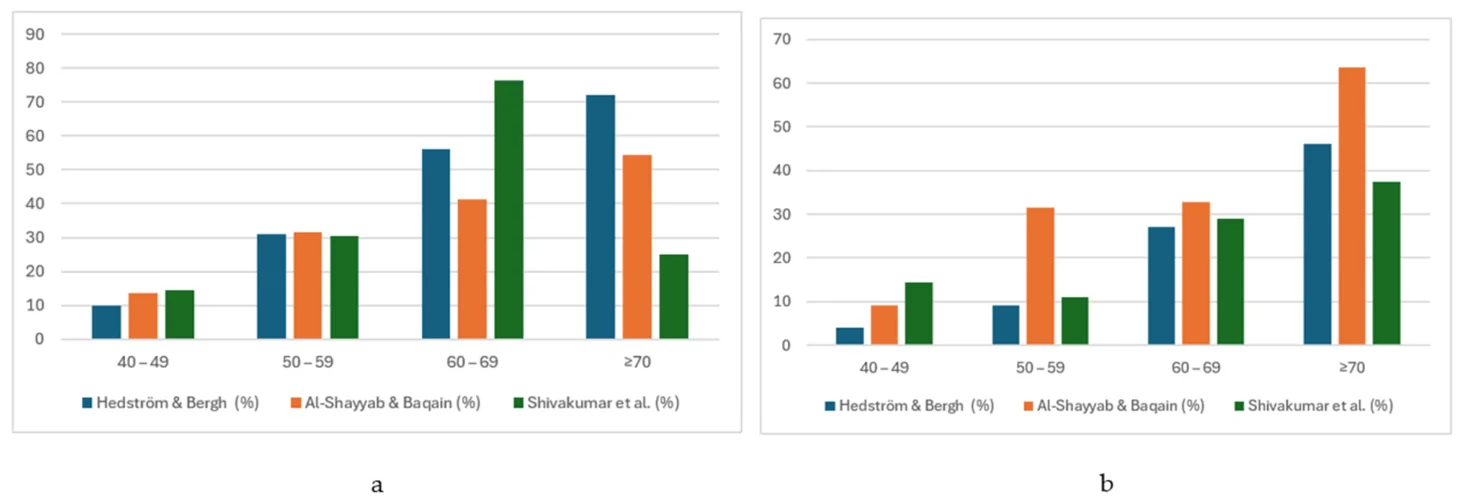
(**a**) Prevalence of LV by age groups in the studies by Hedström and Bergh [14], Al-Shayyab and Baqain [41], and Shivakumar et al. [43] (**b**) Prevalence of CVD by age groups in the studies by Hedström and Bergh [14], Al-Shayyab & Baqain [41], and Shivakumar et al. [43]. Author’s own elaboration based on the included studies.

The prevalence of LV according to age groups was analyzed in the studies by Hedström and Bergh [14], Al-Shayyab and Baqain [41], and Shivakumar et al. [43]. Overall, an increasing trend in the prevalence of LV with age was observed across all three studies (Figure 2a).

In the 40–49 age group, prevalence was low in all studies (≈10–15%). In the 50–59 age group, a moderate increase was observed, with values approaching 30%. This increase became more pronounced in the 60–69 age group, where higher prevalence rates were reached, particularly in the study by Shivakumar et al. [43]. Finally, in the ≥70 age group, the highest values were reported in the studies by Hedström and Bergh [14] and Al-Shayyab and Baqain [41], exceeding 50–70%, while Shivakumar et al. [43] showed a relative decrease compared to the previous age group (Figure 2a).

The prevalence of CVDs by age group was also analyzed in the same studies. A consistent progressive increase in CVD prevalence with age was observed (Figure 2b).

In the 40–49 age group, values were low (<15%). In the 50–59 age group, an increase was observed, particularly in the study by Al-Shayyab and Baqain [41]. In the 60–69 age range, prevalence continued to rise in all three studies (≈25–40%). Finally, from the age of 70 onwards, the highest values were recorded, again highlighted by the study of Al-Shayyab and Baqain [41], with figures exceeding 60% (Figure 2b).

### 3.7. Risk of Bias

The risk of bias of the included studies was assessed using the Joanna Briggs Institute (JBI) tool for cross-sectional studies and the Newcastle–Ottawa Scale (NOS) for cohort studies.

Regarding cross-sectional studies, most presented a low risk of bias. In general, inclusion criteria were clearly defined, and both the characteristics of the population and the study context were adequately described (Table 3). Additionally, exposure measurement was valid and reliable in most studies, and outcomes were assessed using appropriate methodologies. Likewise, most studies identified potential confounding factors and described strategies to control them, which strengthens the internal validity of the findings (Table 3).

As for cohort studies, evaluated using the Newcastle–Ottawa Scale, both showed adequate methodological quality. The study by Bergh et al. [36] achieved a high score, particularly excelling in group comparability and appropriate exposure assessment. Meanwhile, the study by Bergh et al. [37] reached the maximum score in most evaluated domains, indicating a low risk of bias and high methodological quality. In both cases, case definition, participant selection, and exposure verification were appropriately conducted, applying consistent methods to all included subjects (Table 4).

Overall, the analyzed evidence shows a low to moderate overall risk of bias, with a predominance of studies of good methodological quality. The available evidence supports a significant association between the presence of lingual varices (LV) and cardiovascular disease (CVD).

### 3.8. Meta-Analysis

The meta-analysis evaluated the association between the presence of LV and selected cardiovascular and demographic variables.

Pooled effect estimates were calculated using the Mantel–Haenszel (M-H) method. Statistical heterogeneity among studies was assessed using Cochran’s Chi-square test and the I^2^ statistic. A random-effects model was applied when substantial heterogeneity was present (I^2^ > 50%), whereas a fixed-effect model was used when heterogeneity was low (I^2^ ≤ 50%). Statistical significance was assessed using the Z test, with a two-sided *p*-value < 0.05 considered statistically significant. The results are presented as forest plots displaying the individual study estimates and the pooled effect estimates.

Given the variability in HTN definitions across studies, including differences in diagnostic criteria, blood pressure measurement protocols, treatment status, and the reporting of controlled or resistant HTN, studies reporting HTN as a dichotomous variable (presence versus absence of hypertension) were included in the quantitative synthesis. Studies reporting only specific HTN subcategories without comparable dichotomous data were excluded from the HTN meta-analysis. The calculations used for the forest plots and percentage tables are provided in the Supplementary Materials.

**Figure 3:**
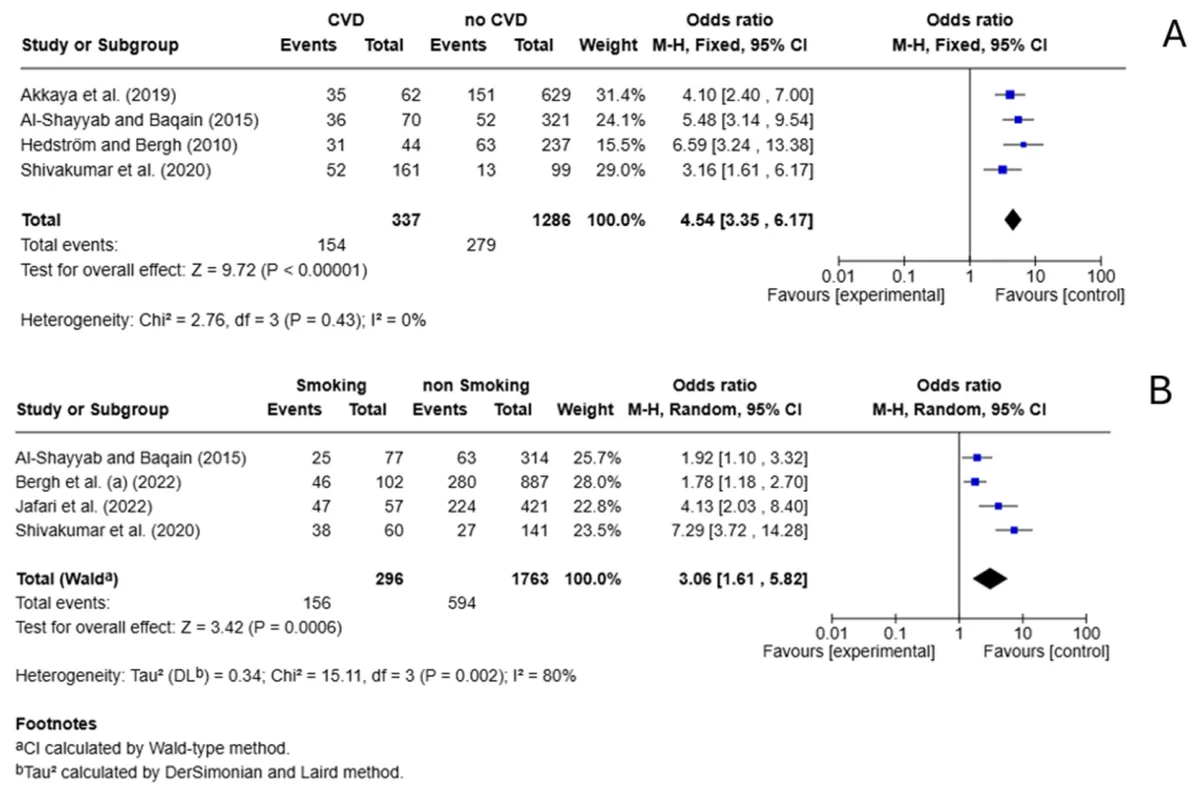
(**A**) Forest plot of the association between the presence of LV and CVD [14,41,42,43]. (**B**) Forest plot of the association between the presence of LV and smoking [33,35,41,43]. Blue squares indicate the point estimates for individual studies, horizontal lines represent the 95% confidence intervals, the black diamond represents the pooled estimate, and the vertical line indicates the line of no effect (OR = 1).

**Figure 4:**
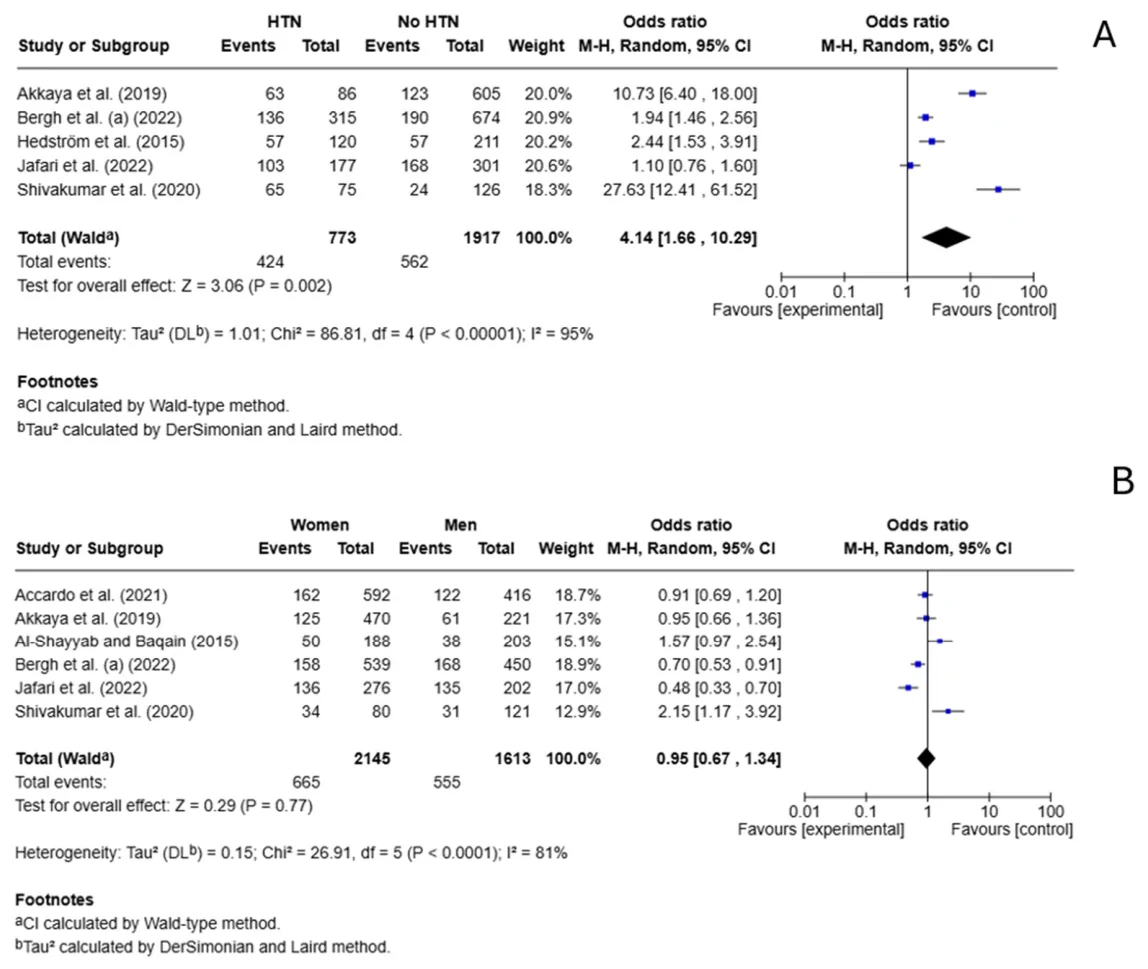
(**A**) Forest plot of the association between the presence of LV and HTN [33,34,35,42,43]. (**B**) Forest plot of the association between the presence of LV and sex [33,35,38,41,42,43]. Blue squares indicate the point estimates for individual studies, horizontal lines represent the 95% confidence intervals, the black diamond represents the pooled estimate, and the vertical line indicates the line of no effect (OR = 1).

The presence of LV was significantly associated with CVD (OR = 4.54; 95% CI: 3.35–6.17; *p* < 0.00001). No evidence of statistical heterogeneity was observed (I^2^ = 0%), indicating highly consistent findings across the included studies (Figure 3A). The presence of LV was significantly associated with HTN (OR = 4.14; 95% CI: 1.66–10.29; *p* = 0.002). Nevertheless, this analysis showed considerable heterogeneity (I^2^ = 95%), suggesting marked variability across studies (Figure 4A).

The presence of LV was significantly associated with smoking (OR = 3.06; 95% CI: 1.61–5.82; *p* = 0.0006). However, substantial heterogeneity was detected (I^2^ = 80%), indicating variability among the included studies (Figure 3B). No significant association was observed between the presence of LV and sex (OR = 0.95; 95% CI: 0.67–1.34; *p* = 0.77). High heterogeneity was also detected (I^2^ = 81%) (Figure 4B).

**Figure 5:**
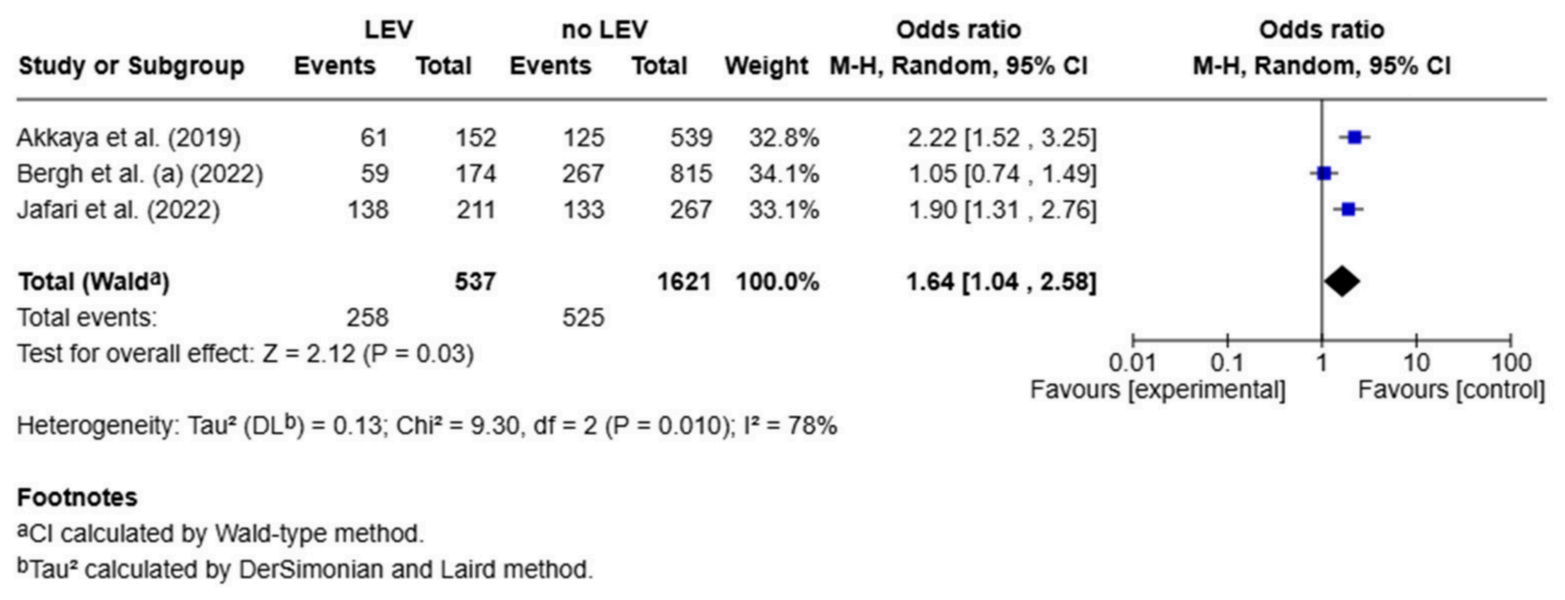
Forest plot of the association between the presence of LV and the presence of LEV [33,35,42]. Blue squares indicate the point estimates for individual studies, horizontal lines represent the 95% confidence intervals, the black diamond represents the pooled estimate, and the vertical line indicates the line of no effect (OR = 1).

The presence of LV was significantly associated with LEV (OR = 1.64; 95% CI: 1.04–2.58; *p* = 0.03). However, substantial heterogeneity was observed (I^2^ = 78%), indicating variability in the magnitude of the association across studies (Figure 5).

However, the pooled estimates for HTN, smoking, sex, and LEV should be interpreted with caution because of the substantial between-study heterogeneity. For these outcomes, the quantitative findings should be considered complementary to the qualitative synthesis rather than definitive summary estimates.

### 3.9. Certainty of Evidence (GRADE)

According to the GRADE approach, the certainty of evidence for the primary meta-analysed outcome was judged to be low.

The certainty of evidence started at low because all included studies were observational. No serious concerns were identified regarding risk of bias, as most studies were assessed as having low to moderate methodological limitations. Likewise, indirectness was not considered serious because the included populations, exposure (LV), and outcome (CVD) directly addressed the review question.

For the primary pooled analysis, inconsistency was not considered serious because no statistical heterogeneity was observed (I^2^ = 0%). However, substantial heterogeneity was identified in several secondary meta-analyses, particularly those evaluating HTN, DM, smoking, LEV, and sex, which should be considered when interpreting those findings.

The certainty of evidence was downgraded for imprecision, owing to the observational nature of the available evidence, the limited number of studies contributing to several pooled analyses, and the methodological variability among studies. Publication bias could not be formally assessed because the number of studies included in several meta-analyses was insufficient for reliable statistical testing, and no clear evidence of publication bias was identified.

Although the pooled estimate demonstrated a large magnitude of association (OR = 4.54; 95% CI: 3.35–6.17), the certainty of evidence was not upgraded because residual confounding and other inherent limitations of observational studies could not be excluded. Overall, the certainty of evidence for the association LV and CVD was therefore rated as low (Table 5).

## 4. Discussion

The findings of the present meta-analysis both support and expand the existing evidence on the association between lingual varices (LV) and cardiovascular diseases (CVD). The qualitative synthesis identified a significant association between LV and CVD which was further supported by the meta-analysis, showing a high association between both conditions (OR = 4.54; 95% CI: 3.35–6.17) with no evidence of statistical heterogeneity. While the review by Lazos et al. [45] concluded that, although LV are common in older individuals, there was no conclusive evidence supporting an association with CVD, a more recent review by the same authors [46] reported significant associations with several systemic conditions, including venous insufficiency, diabetes mellitus (DM), and CVD. The present findings are consistent with this more recent evidence, further supporting an association between LV and CVD. However, the available evidence remains observational and does not support the use of lingual varices as an independent cardiovascular screening marker.

The meta-analytic review by Eslami et al. [15] provides more robust evidence regarding HTN, demonstrating a significant association between LV and hypertension (HTN). Meanwhile, the systematic review by Costa et al. [16] describes an association between LV and several CVD-related factors, such as DM, and smoking, although without providing quantitative estimates of the magnitude of this association with CVD and highlighting the methodological heterogeneity of the included studies. In this context, strengthen the existing evidence supporting an association between LV and CVD, particularly HTN. However, they should be interpreted as evidence of association rather than predictive capability.

Regarding age, it is confirmed as the most consistently associated factor (OR between 1.03 and 3.21) [36,38], with a progressive increase observed in both LV and CVD. This finding aligns with both studies by Lazos et al. [46], which highlight aging as the main associated factor of these lesions.

Additionally, these changes have been proposed to be related to chronic hemodynamic alterations, such as increased venous pressure and reduced vascular elasticity, facilitating the development of venous ectasias [46]. These findings support the hypothesis that LV could be described as a manifestation of physiological vascular aging rather than a primary pathology, although they may be associated with systemic conditions.

The presence of LV showed a relevant association with HTN in individual studies (OR between 1.61 and 2.30) and in meta-analysis (OR = 4.21). In this regard, Lazos et al. [45] suggested that the presence of LV may help identify undiagnosed hypertensive patients. However, this hypothesis requires confirmation in prospective studies evaluating their predictive performance. The meta-analyses by Eslami et al. [15] and Costa et al. [16] also identify it as one of the most relevant factors associated with the presence of LV, although with differing levels of evidence. Eslami et al. [15] report consistent findings, showing a significant association (OR = 2.66) and higher blood pressure values in patients with LV, while Costa et al. [16] show a significant association between HTN and LV (OR = 2.59). Overall, these findings reinforce the consistent association between HTN and the presence of LV, supporting their potential role as a clinical finding associated with systemic vascular alterations.

However, several methodological limitations should be considered when interpreting the present findings. Although most studies were classified as having a low risk of bias, some important weaknesses were identified. In several studies, outcome measurement was only partially adequate because examiner calibration and inter- or intra-examiner agreement were not reported, potentially introducing classification bias [14,33,41,43]. In addition, some studies provided limited or insufficiently detailed statistical analyses [33,44]. Specifically, the studies by Jafari et al. [33] and Dos Santos et al. [44] were judged to have a moderate risk of bias owing to limitations in confounding control and statistical analysis, whereas the study by Ahadian et al. [40] was considered at moderate risk of bias because of deficiencies in outcome measurement, inadequate control of confounding, and insufficient statistical analysis.

Several methodological characteristics of the included studies may also have contributed to the observed heterogeneity. Recruitment settings varied considerably, including public and private dental clinics, university dental hospitals, oral medicine departments, geriatric outpatient clinics, nursing homes, and retrospective pathology archives. Such differences may have influenced the baseline prevalence of both LV and CVD, thereby limiting the generalizability of the pooled estimates to unselected dental populations.

The assessment of LV was not fully standardized across studies, with different clinical grading systems and assessment methods, including direct clinical examination and standardized photography. For the meta-analysis, these classifications were harmonized into comparable dichotomous categories; however, differences in diagnostic criteria may still have influenced case identification. Furthermore, methodological details regarding examiner training, calibration, inter- and intraobserver reliability, examiner blinding, participation rates, missing data, and participant non-response were inconsistently reported, representing additional potential sources of measurement and selection bias. Because only a small number of studies used alternative LV classification systems, it was not possible to perform a meaningful sensitivity analysis according to the classification method. Therefore, the potential influence of different classification systems on the pooled estimates cannot be excluded.

A high level of heterogeneity was observed across several pooled analyses, particularly for HTN, smoking, lower extremities varices (LEV), sex, and DM. This variability is likely explained by methodological and clinical differences among the included studies, including differences in study design, geographic populations, age distribution, diagnostic criteria for lingual varices, and definitions and ascertainment of cardiovascular outcomes. CVD was identified using physician-confirmed diagnoses, medical records, participant self-report, medication history, or blood pressure measurements performed during the study, all of which differ in diagnostic accuracy and may have introduced outcome misclassification.

An important consideration when interpreting the observed associations is the potential influence of residual confounding. Age was the factor most consistently associated with both LV and CVD and is therefore likely to represent an important shared determinant. Likewise, DM, smoking, obesity, and cardiovascular medication use are closely interrelated and frequently coexist. Although several included studies adjusted for some of these variables, adjustment strategies varied substantially, ranging from models including only age or age and sex to more comprehensive multivariable models incorporating smoking, HTN, DM, body mass index, denture wearing, cardiovascular disease, and other clinical variables. Because these adjustment strategies were highly heterogeneous, adjusted effect estimates were not directly comparable and could not be pooled. Consequently, the quantitative synthesis was based on crude dichotomous data to ensure methodological consistency across studies. Although this approach improved comparability, it does not eliminate the possibility of residual confounding, which may have partially influenced the magnitude of the observed associations.

Studies reporting only HTN subcategories without comparable dichotomous data were excluded from the HTN meta-analysis. Nevertheless, residual differences in hypertension assessment may still have contributed to the observed heterogeneity. Similar methodological heterogeneity has also been reported in the meta-analyses by Eslami et al. [15] and Costa et al. [16].

The relatively small number of studies included in each meta-analysis limited the possibility of performing additional analyses, such as prediction intervals, leave-one-out analyses, or influence analyses, and reduced the precision of between-study variance estimates. Likewise, publication bias was not formally assessed because none of the meta-analyses included the minimum of ten studies generally recommended for meaningful funnel plot interpretation. Although some cohort studies were included, separate analyses of incident cardiovascular events were not feasible because follow-up characteristics and time-to-event data were inconsistently reported.

Taken together, these methodological limitations are likely to have influenced the magnitude of the pooled estimates. Therefore, although the overall direction of the association was generally consistent across studies, the strength of the pooled estimates should be interpreted with caution, particularly for outcomes with substantial heterogeneity. For these outcomes, the quantitative findings should be considered alongside the qualitative evidence rather than as definitive summary estimates. Future well-designed prospective studies using standardized diagnostic criteria, objective ascertainment of cardiovascular outcomes, and comprehensive multivariable adjustment are needed to determine whether LV constitute an independent clinical marker of CVD or primarily reflect the cumulative burden of shared risk factors.

Recent studies investigating the lingual microvasculature using advanced imaging techniques provide additional biological support for the observed association between LV and CVD. In particular, a pilot study by Janik et al. [28], based on fractal dimension analysis of sublingual vessels, identified significant differences in vascular morphology between patients with CVD, including HTN, coronary artery disease, atherosclerosis, and valvular heart disease, and healthy controls. These findings suggest that structural alterations in the lingual microvascular network may reflect systemic vascular changes associated with cardiovascular disease and vascular aging, thereby supporting the biological plausibility of the observed association.

In this context, the Spanish study by González-Álvarez and García-Pola [47] provides relevant evidence by analyzing factors associated with lingual lesions using a propensity score–matched case–control design. However, although it identifies associations with systemic factors and medication use, it does not consistently confirm the relationship with CVDs after statistical adjustment, highlighting the possible influence of confounding factors and reinforcing the need for further analytical studies in the Spanish population.

The available evidence mainly originates from different countries, whose populations differ in sociodemographic characteristics, genetic background, and lifestyle factors that may influence the development of LV and their association with cardiovascular diseases. Factors such as dietary habits, the prevalence and management of cardiovascular risk factors, smoking habits, access to healthcare, and population aging may affect both the prevalence of LV and the observed associations with CVD. Furthermore, differences in diagnostic criteria and study methodologies across countries may have contributed to the observed heterogeneity and limit the direct comparability of the available evidence.

On the other hand, it is important to highlight the absence of a standardized classification of LV, as most studies use dichotomous systems (absent/mild vs. moderate/severe), which limits precision in evaluation and comparison of results. Unlike other venous pathologies, such as varicose veins of the lower extremities, where widely accepted classifications allow objective stratification of severity, there is no universally validated system for LV [48]. This lack of standardization hinders comparison between studies, contributes to heterogeneity of results, and highlights the need to develop more homogeneous and reproducible diagnostic criteria and classification systems.

Regarding future research directions, it is necessary to develop studies with more robust methodological designs, especially longitudinal and cohort studies, to establish the temporal relationship between LV and CVD and to determine whether the presence of LV provides incremental clinical value beyond established cardiovascular risk factors. Additionally, it would be of interest to evaluate whether LV have predictive value and diagnostic accuracy sufficient to support their future use as a non-invasive clinical screening tool in dental practice, through prospective studies analyzing their predictive capacity and usefulness in early identification of patients at cardiovascular risk.

Another relevant line of research would be to further explore the role of confounding factors through studies incorporating well-adjusted multivariate analyses and more restrictive models, including variables such as age, smoking, DM, and other cardiovascular systemic factors. This would help determine whether the observed association is independent of or mediated by these factors.

Finally, further research is needed to explore the underlying pathophysiological mechanisms explaining the relationship between LV and CVDs, as well as the potential impact of pharmacological treatments on this association.

Although this review demonstrates a significant association between LV and CVD, association should not be interpreted as predictive capability. The observational nature of the available evidence precludes conclusions regarding the diagnostic accuracy or predictive performance of LV. Prospective studies evaluating sensitivity, specificity, and predictive value are required before considering their implementation as a screening tool in clinical practice.

Furthermore, future studies should determine whether referral strategies based on the presence of lingual varices improve patient outcomes and provide clinically meaningful benefits beyond routine cardiovascular risk assessment, including medical history and blood pressure measurement, rather than merely increasing additional investigations or identifying conditions that would otherwise be detected during standard clinical care.

## 5. Conclusions

The available evidence supports a significant association between the presence of lingual varices (LV) and cardiovascular disease (CVD), particularly hypertension, as well as with smoking. In contrast, no statistically significant associations were observed for sex or lower-extremity varicose veins. However, these findings should be considered inconclusive rather than evidence of the absence of an association because they were based on a limited number of studies, substantial between-study heterogeneity, and wide confidence intervals. Furthermore, owing to the observational nature of the included studies, the considerable heterogeneity across several analyses, and the potential influence of residual confounding, LV should currently be regarded as a clinical finding associated with cardiovascular disease rather than an established cardiovascular screening marker. Further well-designed prospective longitudinal studies are needed to clarify their independent clinical relevance and determine whether they provide incremental value beyond established cardiovascular risk factors.

Due to the observational nature of the included studies, the substantial heterogeneity across several analyses, and the potential influence of residual confounding, LV should currently be regarded as a clinical finding associated with cardiovascular disease rather than an established cardiovascular screening marker. Further prospective longitudinal studies are needed to clarify their clinical relevance.

## Figures and Tables

**Table 2:** (**a**) Characteristics of the included studies: presence and classification of LV, CVDs, CVD- and LV-associated factors, LEV, and medication. (**b**) Characteristics of the included studies: multivariable analysis and variables included. Author’s own elaboration.

(**a**)						
**Author**	**Presence of Lingual Varicose Veins (%)**	**Classification of Lingual Varicose Veins**	**Patients with Cardiovascular Pathologies**	**LV- and CVD-Associated Factors**	**Varicose Veins Lower Extremities**	**Medication**
Hedström and Bergh (2010) [14]	94 (33.4)	G0 (*n* = 187) G1 (*n* = 94)	HTN (*n* = 35; 12.45%) AP (*n* = 5; 1.77%) MI (*n* = 4; 1.42%) Stroke (*n* = 1; 0.35%) AF (*n* = 1; 0.35%) HVD (*n* = 1; 0.35%) CVD and LV (*n* = 31; 11.03%).	Sex (OR = 0.79; *p* = 0.44). Age (OR = 1.11; *p* < 0.0001) Smoking (OR = 2.37; *p* = 0.023)	ND	ND
Hedström et al. (2015) [34]	114 (26.4)	G0 (*n* = 317) G1 (*n* = 114)	HTN (*n* = 120; 27.84%) and LV (*n* = 57; 13.22%)	Age (OR = 1.07; *p* < 0.0001) Smoking (OR = 2.22; *p* = 0.025) HTN (OR = 2.25; *p* = 0.002)	ND	ND
Al-Shayyab and Baqain (2015) [41]	88 (22.5)	G0 (*n* = 303) G1 (*n* = 88)	CVD (*n* = 70; 17.9%) and LV (*n* = 36; 51.4%)	Age (OR = 2.27; *p* = 0.008) Female sex (OR = 2.74; *p* = 0.001) Smoking (OR = 2.93; *p* = 0.002) URD (OR = 2.03; *p* = 0.044) CVD (OR = 4.01; *p* < 0.001)	ND	ND
Akkaya et al. (2019) [42]	186 (26.9)	G0 (*n* = 505) G1 (*n* = 186)	CVD (*n* = 62; 8.97%) and LV (*n* = 35; 5.06%) HTN (*n* = 86; 12.44%) and LV (*n* = 63; 9.12%)	HTN (OR = 2.30; *p* = 0.007) URD (OR = 2.17; *p* = 0.020)	LEV (*n* = 152; 22%) and LV (*n* = 61; 8.82%)	ND
Shivakumar et al. (2020) [43]	65 (32.33%).	G0 (*n* = 112) G1 (*n* = 89)	HTN (*n* = 75; 37.31%) and LV (*n* = 45; 22.38%) MI (*n* = 9; 4.47%) and LV (*n* = 2; 0.99%) AF (*n* = 13; 6.46%) and LV (*n* = 2; 0.99%) Stroke (*n* = 5; 2.48%) and LV (*n* = 1; 0.49%) IHD (*n* = 7; 3.48%) and LV (*n* = 2; 0.99%)	Age (OR = 3.21) Female sex (OR = 2.89) Smoking (OR = 3.13) CVD (OR = 3.97)	ND	ND
Accardo et al. (2021) [38]	284 (28.17%)	G0 (*n* = 724) G1 (*n* = 284)	HTN (*n* = 624; 61.9%) New HTN and LV (*n* = 25; 2.48%) Controlled HTN and LV (*n* = 133; 13.2%) Resistant HTN with LV (*n* = 69; 6.84%)	Age (*p* < 0.001) HTN Compensation (*p* < 0.005) Resistant HTN (*p* < 0.005) DL (*p* < 0.05) Smoking (*p* < 0.05) Sex (*p* ≥ 0.05) Antihypertensive therapy: (*p* < 0.05)	ND	No significant association described in multivariate analysis.
Bergh et al. (2022a) [35]	326 (32.96%)	G0 (*n* = 663) G1 (*n* = 326)	HTN (*n* = 315; 31.85%) and LV (*n* = 136; 13.75%)	HTN (OR = 1.61; *p* = 0.001) Systolic pressure ≥140 mmHg (OR = 1.51; *p* = 0.014) DM (OR = 1.8; *p* < 0.05) DL (OR = 0.78; *p* = 0.168) Raised waist-to-hip ratio (OR = 2.06; *p* = 0.004).	LEV (*n* = 174; 17.6%) and LV (*n* = 59; 5.96%)	ND
Baharvand et al. (2022) [39]	91 (60.26%)	G0 (*n* = 60) G1 (*n* = 68) G2 (*n* = 23)	HTN (*n* = 37; 24.5%) and LV (*n* = 33; 21.85%)	Age (OR = 1.03; *p* = 0.052) Male sex (OR = 2.07; *p* = 0.053) Smoking (OR = 0.50; *p* = 0.12)	ND	ND
Bergh et al. (2022b) [36]	86 (30.6%)	G0 (*n* = 195) G1 (*n* = 86)	HTN (*n* = 90; 32%) and LV (*n* = 14; 4.98%) CVD (*n* = 21; 7.47%) and LV (*n* = 5; 1.77%)	Elderly age (*p* = 0.003) Sex (*p* = 0.115) Smoking (*p* = 1.000) DM-II (*p* = 0.517) DL (*p* = 0.658) CVD (*p* = 0.307) BMI (*p* = 0.374) HTN (*p* = 0.891)	ND	ND
Jafari et al. (2022) [33]	271 (56.69%)	ND	HTN (≥140/90 mmHg) (*n* = 177; 37.02%) and LV (*n* = 103; 21.54%)	Male gender (*p* < 0.001) Age (*p* < 0.01) Smoking (*p* < 0.001) URD (*p* < 0.01) LEV (*p* < 0.0001)	LEV (*n* = 211; 44.14%) and LV (*n* = 138; 28.87%).	ND
Ahadian et al. (2023) [40]	84 (70%)	G0 G1 G2	HTN (*n* = 68; 56.6%) and with LV (*n* = 59; 86.4%)	Age (*p* < 0.001) Sex (*p* = 0.549)	ND	ND
Bergh et al. (2024) [37]	377 (33.1%)	G0 G1	HTN (*n* = 374; 32.83%) and with LV (*n* = 168; 14.75%) Previous events of total IHD (*n* = 69; 6.05%) and LV (*n* = 38; 3.33%) Previous total cerebrovascular events (*n* = 66; 5.79%) and LV (*n* = 29; 2.55%) New events of total IHD (*n* = 31; 2.72%) and LV (*n* = 17; 1.49%) New total cerebrovascular events (*n* = 31) and LV (*n* = 16)	New ischemic heart disease events (OR = 2.26): Age (OR = 1.57; *p* < 0.0001) Sex (OR = 0.63; *p* < 0.0001) Smoking (OR = 2.08; *p* < 0.0001) New cerebrovascular events (OR = 1.77): Age (OR = 1.56; *p* < 0.0001) Sex (OR = 0.62; *p* < 0.0001) Smoking (OR = 2.01; *p* < 0.0001)	LEV (*n* = 210; 18.44%) and LV (*n* = 72; 6.32%).	ND
Dos Santos et al. (2024) [44]	134 (83.22%)	ND	HTN and LV (*n* = 73; 45.34%) Total MI and LV (*n* = 45; 27.95%) Total thrombosis (*n* = 41; 25.46%) Total AP (*n* = 2; 1.24%) Total stroke (*n* = 4; 2.48%)	HTN (*p* = 0.184) DM (*n* = 107; *p* < 0.001) MI (*p* = 0.012) Thrombosis (*n* = 41; *p* = 0.004)	ND	Losartan (*n* = 11) Simvastatin (*n* = 6) Hydrochlorothiazide (*n* = 5) Metformin (*n* = 3) NSAIDs and antidepressants in smaller proportions.
(**b**)						
**Author**		**Multivariable Analysis** **(Yes/No)**	**Variables Included in the Multivariable Model**			
Hedström y Bergh (2010) [14]		Yes	Age, sex, smoking, and cardiovascular disease (CVD). Logistic regression with sublingual varices as the dependent variable.			
Hedström et al. (2015) [34]		Yes	Age, smoking, and HTN, with the model adjusted CVD and LEV.			
Al-Shayyab and Baqain (2015) [41]		Yes	Age, sex, smoking, URD, dietary preference (vegetables vs. others), and CVD.			
Akkaya et al. (2019) [42]		Yes	Variables entered into the multivariable logistic regression were age, smoking, systemic diseases, HTN, other CVD, DM, LEV, URD. In the final model, only age, HTN and URD remained statistically significant.			
Shivakumar et al. (2020) [43]		Yes	Age, sex, smoking, smoking duration, and medical history (CVD vs. non-CVD).			
Accardo et al. (2021) [38]		Yes	Age, HTN (normotensive, newly diagnosed, compensated or resistant), smoking, DM, DL, obesity, antihypertensive therapy, and sex. Stepwise selection identified age, HTN status, and antihypertensive treatment as the variables retained for the final multivariable regression tree model.			
Bergh et al. (2022a) [35]		Yes	Separate multivariable logistic regression models adjusted for age and sex (Model 1) and age, sex and smoking (Model 2). Independent variables evaluated included HTN, systolic blood pressure, fasting glucose/DM2, DL (cholesterol and HDL), BMI, waist circumference and waist-to-hip ratio.			
Baharvand et al. (2022) [39]		Yes	Logistic regression adjusted for age, sex, and smoking. The model evaluated the association between grade of sublingual varicosity (grades 0, 1, and 2) and HTN while controlling for these confounders. The model also estimated the effects of sex and smoking on the presence of LV.			
Bergh et al. (2022b) [36]		Yes	Logistic regression (enter model) adjusted for age and sex. The model assessed whether CVD and other cardiovascular-associated factors (DM2, DL, BMI, smoking and snuff use) were associated with the development or persistence of LV over the 8-year follow-up.			
Jafari et al. (2022) [33]		No	Univariate analyses only (Chi-square): age, sex, smoking, HTN, URD, partial denture, LEV and literacy level.			
Ahadian et al. (2023) [40]		No	Univariate analyses only (Chi-square): age, sex and HTN.			
Bergh et al. (2024) [37]		Yes	Multiple logistic regression adjusted for age (categorised), sex and smoking. The models evaluated the association between LV and new IHD and new cerebrovascular disease (stroke/TIA).			
Dos Santos et al. (2024) [44]		No	Univariate analyses only (Chi-square and Fisher’s exact tests): HTN, DM, MI and thrombosis.			

AF, atrial fibrillation; AP, angina pectoris; BMI, body mass index; CVD, cardiovascular disease; DL, dyslipidemia; DM, diabetes mellitus; DM-II, type 2 diabetes mellitus; G0, absence of lingual varicose veins; G1, presence of lingual varicose veins; G2, severe lingual varicose veins; HTN, hypertension; HVD, heart valve disease; IHD, ischemic heart disease; LEV, lower extremity varicose veins; LV, lingual varicose veins; MI, myocardial infarction; ND, not described; NSAIDs, non-steroidal anti-inflammatory drugs; OR, odds ratio; *p*, *p*-value; Stroke, cerebrovascular accident; URD, upper removable denture.

**Table 3:** Assessment of the risk of bias of cross-sectional studies using the JBI Appraisal Tool.

Study	Were the Inclusion Criteria in the Sample Clearly Defined?	Were the Study Subjects and the Setting Described in Detail?	Was the Exposure Measured in a Valid and Reliable Way?	Were Objective, Standard Criteria Used for Measurement of the Condition?	Were Confounding Factors Identified?	Were Strategies to Deal with Confounding Factors Stated?	Were the Outcomes Measured in a Valid and Reliable Way?	Was Appropriate Statistical Analysis Used?	Results
Hedström y Bergh (2010) [14]	Yes	Yes	Yes	Yes	Yes	Yes	Unclear	Yes	Low risk
Hedström et al. [34]	Yes	Yes	Yes	Yes	Yes	Yes	Yes	Yes	Low risk
Al-Shayyab and Baqain [41]	Yes	Yes	Yes	Yes	Yes	Yes	Unclear	Yes	Low risk
Akkaya et al. [42]	Yes	Yes	Unclear	Yes	Yes	Yes	Yes	Yes	Low risk
Shivakumar et al. [43]	Yes	Yes	Yes	Yes	Yes	Yes	Unclear	Yes	Low risk
Accardo et al. [38]	Yes	Yes	Yes	Yes	Yes	Yes	Yes	Yes	Low risk
Bergh et al. [35]	Yes	Yes	Yes	Yes	Yes	Yes	Yes	Yes	Low risk
Baharvand et al. [39]	Yes	Yes	Unclear	Yes	Yes	Yes	Yes	Yes	Low risk
Jafari et al. [33]	Yes	Yes	Yes	Yes	Yes	No	Unclear	Unclear	Moderate risk
Ahadian et al. [40]	Yes	Yes	Yes	Yes	Yes	No	Unclear	Unclear	Moderate risk
Dos Santos et al. [44]	Yes	Yes	Yes	Yes	Yes	No	Yes	Unclear	Moderate risk

Each item was rated as ‘Yes’, ‘No’, or ‘Unclear’. ‘Yes’ indicated that the methodological criterion was fulfilled, ‘Unclear’ indicated insufficient information or partial fulfilment, and ‘No’ indicated that the criterion was not fulfilled. The overall risk of bias was classified as low (7–8 ‘Yes’ responses), moderate (5–6 ‘Yes’ responses), or high (≤4 ‘Yes’ responses).

**Table 4:** Assessment of the risk of bias of cohort studies using the Newcastle–Ottawa Scale (NOS).

Study	Representativeness of the Exposed Cohort	Selection of the Non-Exposed Cohort	Ascertainment of Exposure	Demonstration That Outcome of Interest Was Not Present at Start of Study	Comparability of Cohorts on the Basis of the Design or Analysis Controlled for Confounders	Assessment of Outcome	Was Follow-Up Long Enough for Outcomes to Occur	Adequacy of Follow-Up of Cohorts	Results
Bergh et al. [36]	★	★	★	★	★★	★	★	★	Good Quality
Bergh et al. [37]	★	★	★	★	★★	★	★	★	Good Quality

★ Indicates that the corresponding item was satisfied. ★★ Indicates the maximum score awarded in the comparability domain. No star: item not satisfied or insufficient information available. Good quality: 3 or 4 stars in selection domain AND 1 or 2 stars in comparability domain AND 2 or 3 stars in outcome/exposure domain. Fair quality: 2 stars in selection domain AND 1 or 2 stars in comparability domain AND 2 or 3 stars in outcome/exposure domain Poor quality: 0 or 1 star in selection domain OR 0 stars in comparability domain OR 0 or 1 stars in outcome/exposure domain.

**Table 5:** GRADE assessment of the certainty of evidence for the association between lingual varices and cardiovascular disease.

Outcome	No. Studies	Study Design	Risk of Bias	Inconsistency	Indirectness	Imprecision	Publication Bias	Overall Certainty
Association between lingual varices and cardiovascular disease	13	Observational studies	Not serious	Not serious	Not serious	Serious	Undetected	Low

## Data Availability

All data generated or analyzed during this study are included in this published article. The review protocol was prospectively registered in PROSPERO (registration number CRD420251172737).

## Supplementary Materials

The following supporting information can be downloaded at: https://www.mdpi.com/article/10.3390/jcm15166495/s1, File S1: Supplementary Table S1; File S2: PRISMA 2020 checklist.

## Abbreviations

The following abbreviations are used in this manuscript:

LV	Lingual varices
CVD	Cardiovascular diseases
SV	Sublingual varices (moderate/severe)
nSV	No or mild sublingual varices
HTN	Hypertension
DM	Diabetes mellitus
OR	Odds ratio
95% CI	95% confidence interval
M-H	Mantel–Haenszel
BMI	Body mass index
PRISMA	Preferred Reporting Items for Systematic Reviews and Meta-Analyses
JBI	Joanna Briggs Institute
NOS	Newcastle–Ottawa Scale
PECO	Population, Exposure, Comparison, Outcome
ICD-11	International Classification of Diseases, 11th Revision
WHO	World Health Organization
IDF	International Diabetes Federation
RevMan	Review Manager (Cochrane Collaboration software)
